# Design and Development of Levodopa Loaded Polymeric Nanoparticles for Intranasal Delivery

**DOI:** 10.3390/ph15030370

**Published:** 2022-03-18

**Authors:** Mohd Zulhelmy Ahmad, Akmal Hidyat Bin Sabri, Qonita Kurnia Anjani, Juan Domínguez-Robles, Normala Abdul Latip, Khuriah Abdul Hamid

**Affiliations:** 1 Department of Pharmaceutics, Faculty of Pharmacy, Universiti Teknologi MARA Cawangan Selangor, Puncak Alam 42300, Malaysia; zulhelmy88@gmail.com; 2 School of Pharmacy, Queen’s University Belfast, Medical Biology Centre, 97 Lisburn Road, Belfast BT9 7BL, UK; a.binsabri@qub.ac.uk (A.H.B.S.); qanjani01@qub.ac.uk (Q.K.A.); j.dominguezrobles@qub.ac.uk (J.D.-R.); 3 Atta-ur-Rahman Institute for Natural Product Discovery (AuRINS), Universiti Teknologi MARA Cawangan Selangor, Puncak Alam 42300, Malaysia; drnormala6351@uitm.edu.my

**Keywords:** nanoparticles, chitosan, PLGA, bioavailability

## Abstract

Intranasal delivery is an alternative administration route to deliver levodopa (L-Dopa) to the brain. This drug delivery route offers high drug permeability across the nasal epithelium and rapid absorption into the central nervous system (CNS) while bypassing first-pass metabolism. In this study, we developed a library of polymeric nanocarrier systems for L-Dopa utilising poly(lactic-co-glycolic acid) (PLGA) and chitosan. A total of three PLGA nanoparticles formulations (P1, P2 and P3) were prepared using a modified water-in-oil-in-water (W/O/W) solvent evaporation technique, while four formulations of chitosan nanoparticles (C1, C2, C3 and C4) were prepared by ionic gelation method with sodium tripolyphosphate (TPP) as a cross-linking agent. Upon characterising nanocarriers developed, it was discovered that C2 demonstrated the best results with regard to droplet size (553 ± 52 nm), polydispersity index (0.522), zeta potential (+46.2 ± 2.3 mV), and encapsulation efficiency (82.38% ± 1.63). Transmission electron microscopy (TEM) and scanning electron microscopy (SEM) further corroborated the particle size analysis highlighting that C2 displayed uniform particle size with spherical morphology. Additionally, X-ray diffraction analysis (XRD) revealed that C2 was in an amorphous state while Fourier transform infrared (FTIR) analysis showed that there were no chemical interactions that might change the chemical structure of L-Dopa within the polymeric nanoparticle matrix. Lastly, an in-vivo intranasal study in male Wistar rats showed that the absorption of L-Dopa when formulated as chitosan nanoparticles was significantly enhanced (*p* < 0.05) by approximately two-fold compared to unmodified L-Dopa. Therefore, this work illustrates that formulating L-Dopa into chitosan nanoparticles for intranasal delivery is a potentially viable formulation strategy to improve the bioavailability of the drug for the treatment of Parkinson’s disease.

## 1. Introduction

Levodopa (L-Dopa) is a medication used to treat Parkinson’s disease (PD), which is a condition associated with low levels of a chemical called dopamine in the brain [1]. Unlike dopamine, L-Dopa is able to cross the BBB by utilising the large neutral amino acid transporter (LAT1), which is expressed on the endothelial cells present on this border. Upon crossing the BBB, L-Dopa is then regionally metabolised via decarboxylation to dopamine [2]. L-Dopa can be administered orally, but less than 1% of the administered dose reaches the CNS due to the rapid metabolism of dopa decarboxylase (DDC) and catechol-o-methyltransferase (COMT) that occurs during hepatic first-pass metabolism [3]. Although the oral route offers a large surface area for absorption along the small intestine, hydrophilic drugs have a high risk of premature degradation due to rapid metabolism within the gastrointestinal tract (GIT) coupled with hepatic first-pass metabolism [4].

In order to overcome the issues associated with premature peripheral metabolism of L-Dopa, the drug is frequently co-administered with the DDC inhibitor carbidopa. However, co-administration of L-Dopa with carbidopa has been reported to induce a range of unwanted side effects such as leg pain, ataxia and increased tremor, which may affect the patient’s overall quality of life. In addition, oral administration of Parkinson’s drugs is quite challenging as up to 80% of patients with the disease tend to suffer some form of dysphagia, thus necessitating healthcare professionals to explore alternative routes of administration [5]. Given this limitation, intranasal administration offers an alternative delivery route for administering L-Dopa with acceptable bioavailability and avoiding the need for carbidopa co-administration. The nasal epithelium is a highly permeable monolayer that contains a highly vascularized submucosa conferring an optimal site for drug absorption [6].

The interest in developing a suitable formulation capable of delivering L-Dopa via intranasal is increasing. In addition, efforts have been made to improve the rate and extent of drug transport across the membrane, which includes alteration of the lipophilicity of the drug [7], reformulation of the drug to achieve higher water solubility [8], and delivery of the encapsulated drugs within biodegradable polymeric nanoparticles [9]. In addition, delivering a drug through the nasal route circumvents hepatic first-pass metabolism, an issue typically associated with oral administration of L-Dopa. Nevertheless, the intranasal route suffers from the issue of mucociliary clearance along the nasal surface. Mucociliary clearance or mucociliary transport is an innate self-clearing mechanism of the nasal airways that will dismiss and expel any exogenous material via coughing and sneezing [10]. One way to mitigate this natural biological response is to reformulate drugs into nanoparticulate delivery systems with nose-to-brain targeting properties to enable targeted delivery of therapeutic along the nasal passage. In order to achieve this, the nanoparticulate system ought to exhibit mucoadhesive properties to help minimise the impact of mucociliary clearance. This would enable longer residence time within the nasal mucosa to enable more rapid uptake into systemic circulation hence improving the bioavailability of the drug. One of the polymers that has been investigated in the manufacture of mucoadhesive drug delivery systems is chitosan which is a cationic linear polysaccharide derived from chitin through *N*-deacetylation [11]. Along with its mucoadhesive properties and its effects on epithelial membranes permeability, chitosan nanoparticles are deemed a promising formulation for intranasal delivery [12]. Although several papers detailing chitosan and PLGA nanoparticles loaded with levodopa for intranasal delivery have been reported in the literature, most of these systems typically present with low entrapment efficiency (≈40%) [13,14,15]. Such low entrapment efficiency would result in considerable drug wastage during formulation fabrication while necessitating high doses of nanoparticles to be delivered in order to achieve a sufficient therapeutic level within systemic circulation. Therefore, there is a need to refine and improve these nanocarriers for nose-to-brain delivery purposes.

In this study, we present the development, characterisation and evaluation of different polymeric nanoparticles with the aim of enhancing the entrapment efficiency along with the pharmacokinetic profiles of L-Dopa for intranasal delivery. The nanoparticles were formulated using two types of polymers, PLGA and chitosan. PLGA nanoparticles (formulation P1, P2 and P3) were prepared using a solvent evaporation technique, while chitosan nanoparticles (formulation C1, C2, C3 and C4) were prepared by ionic gelation method with sodium tripolyphosphate (TPP) as a cross-linking agent to entrap the payload, L-dopa. A series of experiments were performed to characterise the particle size distribution, zeta potential, morphology and drug content of the polymeric nanoparticles. Moreover, an in vivo study was conducted to evaluate the pharmacokinetic profile of L-Dopa nanoparticles relative to drug solution following intranasal administration. Therefore, the current work can serve as a basis for future clinical studies that could utilise the system for the delivery of other antiparkinson drugs used to manage the disease in a simple and non-invasive manner.

## 2. Materials and Methods

### 2.1. Materials

Low molecular weight chitosan (50–60 kDa), a mucoadhesive polymer, sodium tripolyphosphate (TPP) was used as a cross-linking agent, and poly (lactic-co-glycolic acid) (PLGA) with a ratio of 50:50 were obtained from Sigma Aldrich (St. Louise, MO, USA). Tween^®^ 80 and dichloromethane (DCM) were purchased from Fisher Scientific (Waltham, MA, USA). Polyvinyl alcohol (PVA) and acetic acid were supplied from R&M Chemicals (Essex, UK). Tetrahydrofuran (THF) HPLC grade and methanol were obtained from Merck (Darmstadt, Germany). Trifluoroacetic acid (TFA) was purchased from Scharlau (Sentmenat, Spain). L-Dopa was supplied from Zhejiang Wild Wind Pharmaceutical Co. Ltd. (Dongyang, China) and used as a model drug. Deionised water (DI) was obtained from Reservoir^®^ Elga Water System (High Wycombe, UK). All other chemicals and solvents were of analytical grade.

### 2.2. Preparation of L-Dopa Loaded PLGA Nanoparticles

**Figure 1:**
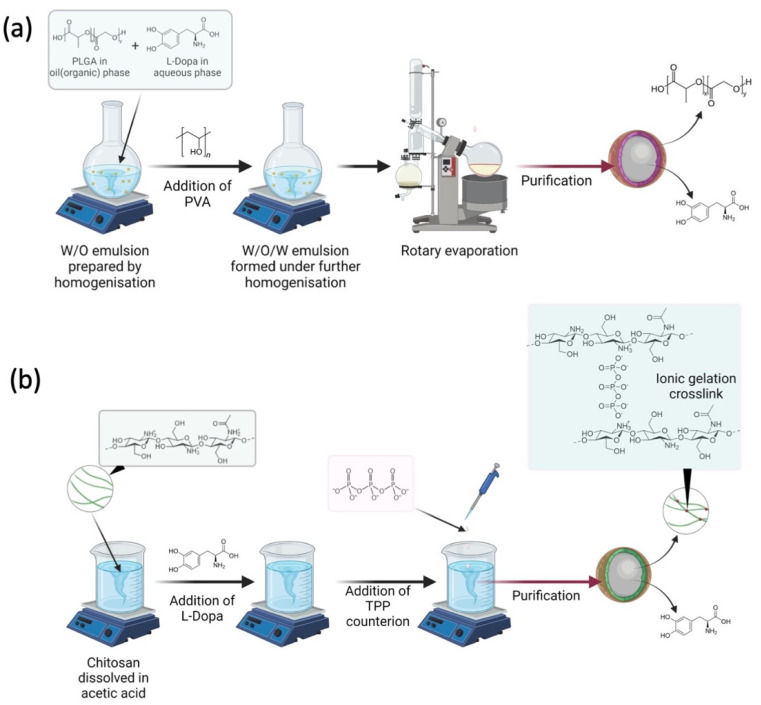
(**a**) Schematic illustrating how L-Dopa loaded PLGA nanoparticles were prepared via water-in-oil-in-water (W/O/W) modified solvent evaporation technique. (**b**) Schematic illustrating how L-Dopa loaded PLGA nanoparticles were prepared via ionic gelation.

L-Dopa loaded PLGA nanoparticles were prepared by the water-in-oil-in-water (W/O/W) modified solvent evaporation technique, as illustrated in Figure 1a [16]. We tried other PLGA in our preliminary work, such as those with a ratio of 65:35 of lactide and glycolide; however, the combination of L-Dopa and this polymer produced a bigger particle size as compared to PLGA with a ratio of 50:50. Therefore, we decided to work with a 50:50 lactide and glycolide PLGA polymer to develop L-Dopa loaded PLGA nanoparticles. Firstly, 10 mg of L-Dopa was dissolved in 10 mL distilled water to form an aqueous phase. Then, 3 different concentrations of PLGA (1.25, 2.5, and 5.0 mg/mL) were prepared in methylene chloride before being homogenised with the drug containing aqueous phase at 22,000 rpm for 30 s using Ultra-Turrax T25 homogeniser (IKA-Labtechnik, Staufen, Germany) to form water-in-oil (W/O) emulsion. Following that, 25 mL of 0.2% PVA was added into the emulsion and then homogenised for 3 min before being submerged in an iced water bath containing ice cubes to form water in the W/O/W emulsion. The W/O/W emulsion was then placed on a rotary evaporator (Buchi, Flawil, Switzerland) and evaporated under reduced pressure until 3–4 mL of the suspension was obtained. Subsequently, the nanoparticles were separated from the suspension by centrifugation at 12,000 rpm for 15 min. Finally, the nanoparticles were lyophilised and stored until further analysis. The drug entrapment efficiency (EE) was calculated by collecting and analysing the supernatant as detailed in Section 2.4.2. This experiment was conducted in order to elucidate the effect of PLGA composition on nanoparticle size and properties. Table 1 summarises the different PLGA nanoparticles formulation manufactured.

### 2.3. Preparation of L-Dopa Loaded Chitosan Nanoparticles

Chitosan nanoparticles were prepared using the ionic gelation method, which involved an ionic cross-linking reaction of chitosan solution with sodium TPP in Tween^®^ 80 as a resuspending agent to avoid particle aggregation. The study was done at ambient temperature under stirring, as previously reported (Figure 1b) [17]. Chitosan was dissolved in 10 mL acetic acid at different concentrations of 0.1, 0.2, 0.3, and 0.4% *w/v* before 0.5% *w/v* Tween^®^ 80 was added to the solution. Following that, 10 mg of L-Dopa was added to the chitosan solution. Finally, 0.1% *w/v* sodium TPP, a cross-linking agent, was introduced into the chitosan solution to initiate cross-linking, which then led to the formation of L-Dopa loaded chitosan nanoparticles. The solutions were then centrifuged at 12,000 rpm for 30 min before being lyophilised for further analysis, while the supernatant was collected and analysed for drug EE as detailed in Section 2.4.2. This experiment was conducted in order to elucidate the effect of chitosan composition on nanoparticle size and properties. Table 2 shows the formulation composition for L-Dopa loaded chitosan nanoparticles.

### 2.4. Characterisation of Nanoparticles

#### 2.4.1. Particle Size Analysis

The particle size of the nanoparticles was analysed using Malvern Mastersizer Hydro 2000 (Malvern Instruments, Worcestershire, UK) particle size analyser. All formulations were tested for the size and uniformity distribution. Firstly, the lyophilised nanoparticles were dispersed in 10 mL distilled water. Then, the samples were loaded into the sample chamber using the dropper in a dropwise manner until the laser diffraction of the instrument reached 0.2 units of uniformity before the reading could be started. Measurements were recorded and calculated using the Mastersizer software to obtain the mean value and to ensure the consistency and reproducibility of the results.

#### 2.4.2. Drug Entrapment Efficiency

The amount of L-Dopa entrapped within the nanoparticles was determined by measuring the amount of non-entrapped drug in the supernatant recovered after centrifugation by UV/Vis spectrophotometer (Hitachi Co., Tokyo, Japan). A complete UV/Vis scan was performed to determine the wavelength for maximum absorption of L-Dopa. It was found that L-Dopa displayed a maximum absorption wavelength at 280 nm. Thus, the drug content was measured spectrophotometrically at 280 nm after a calibration curve was constructed. Supernatant collected from nanoparticles preparation was measured to determine the amount of unbound drug. The EE was calculated based on Equation (1):

(1) $$E\;\left(\%\right)=\frac{Total\;amount\;of\;drug-Total\;amount\;of\;unbound\;drug}{Total\;amount\;of\;drug}\times 100\%$$

#### 2.4.3. Zeta Potential

The zeta potential of the nanoparticles was measured by the Malvern Zetasizer 1600 Nano ZS (Malvern Instruments, Worcestershire, UK). First, 1 mg of the nanoparticles was resuspended in 10 mL of deionised water before measurement. The suspension was then introduced into the folded capillary cell DTS1060 (Malvern Instruments, Worcestershire, UK) using a 3 mL syringe until the electrodes of the cell were fully immersed. This was done carefully to mitigate any air bubble formation during sample loading. The cell was then placed into the Zetasizer and equilibrated at 20 °C for 2 min prior to the measurement. The values were recorded by calculating the mean value of 3 samples at 25 °C with a detection angle of 90°.

#### 2.4.4. Morphology Study

Transmission electron microscopy (TEM) analysis of the prepared nanoparticulate formulations was carried out to elucidate the morphology of the nanoparticles. Firstly, nanoparticles were dispersed in distilled water to produce nanosuspension. A drop of nanoparticles suspension was then placed on a carbon film and coated with copper on a TEM grid. Following that, TEM studies were performed at 200 kV using FEI TECNAI G2 20S (TWIN OR, USA. The TEM grid was fixed into a sample holder and placed in a TEM vacuum chamber and observed under low vacuum before TEM images were recorded.

In addition to TEM, the particle size of the nanoparticles was photographed using a scanning electron microscope (SEM) for the morphology analysis. SEM (FEI Quanta 200, Eindhoven, Netherlands) was used in this study due to its high resolution and simple operation, employing a field emission gun for the electron source. First, each of the nanoparticle formulations was transferred onto a 20 × 20 mm glass slide and mounted on an aluminium stub using double-sided carbon tape. The solution was slowly evaporated at room temperature. The completely dried samples were coated with platinum by sputter coating. The image was then captured on a digital microscope at the desired magnification. The photographs were captured at an accelerating voltage of 15 kV, and the mean particle sizes of the nanoparticles were determined using Submicron Particle Sizer software.

#### 2.4.5. X-ray Powder Diffraction Analysis

X-ray powder diffraction (XRD) was performed on formulation C2 because this formulation demonstrated good particle size and uniformity while displaying the highest EE relative to other formulations. Diffractograms obtained was used to determine the solid state of the drug within the nanoparticles. A Ni-filtered Cu Kα radiation over the 2θ range of 10–90° was used in the instrument setting. Samples were placed on a glass substrate, and the experimental parameters were set at a voltage of 40 kV, current at 20 mA, and angular speed at 4°/min, respectively. The diffractograms of blank chitosan, L-Dopa, and L-Dopa loaded chitosan nanoparticles (formulation C2) were recorded and compared.

#### 2.4.6. Fourier Transform Infrared (FTIR) Analysis

FTIR analysis was employed to understand any excipient drug and interaction that may arise within the nanoparticle formulation. For the FTIR study, only formulation C2 was analysed because this formulation demonstrated good particle size and uniformity while displaying the highest EE relative to other formulations. The spectrum of FTIR was recorded in the region of 4000 to 500 cm^−1^. The sample was mixed with potassium bromide and KBr using mortar and pestle. The mixture of samples with KBr was then collected and inserted into a pellet mould to be pressed into pellet form using a hand press. After the sample and potassium bromide had been pressed, the thin pellet formed was then inserted into the FTIR spectrometer. The background emission spectrum of the IR source was first recorded, followed by the emission spectrum of the IR source with the sample in place.

#### 2.4.7. Instrumentation and Chromatographic Condition for the Analytical Method

The HPLC method for quantifying the amount of L-Dopa present rat plasma was developed and validated with respect to linearity, recovery, specificity, accuracy, precision, and stability. All parameters were within the limit as proposed by USFDA guidelines. The proposed method has been published and was designed for rapid quantification of L-Dopa in rat plasma and [18]. Briefly, the samples were treated with a deproteinising agent (DA) consisting of acetonitrile:propanol (1:1) at the ratio of 2:1 (DA: samples) to remove the protein. Then, the mixture was vortexed for 1 min and centrifuged again at 10,000 rpm for 10 min. Prior to analysis, 50 µL of the clear supernatant was filtered and injected onto the HPLC system for chromatographic analysis. Blank plasma was also pre-treated with DA as samples before being injected onto the HPLC system. Due to the potential of photo-degradation of L-Dopa, all the works were performed in a dark environment to reduce exposure to light and in a triplicate manner. Agilent HP 1200 coupled with a diode array detector and evaporative light scattering detector (Agilent Technologies, Santa Clara, CA, USA) was used for analysis. Phenomenex reversed-phase C18 column, Jupiter 5u C18 300 A with a particle size of 5 µm (150 mm × 4.6 mm i.d.) was used for the chromatographic separation. The mobile phase comprised of a mixture of water containing 0.1% trifluoroacetic acid and tetrahydrofuran with the ratio of (97:3) was used in this study. The analysis was done using the isocratic technique with a flow rate of 1.0 mL/min. Prior to HPLC analysis, the mobile phase was filtered through a nylon membrane with 0.45 mm pore size to eliminate any contaminants. The eluent was monitored with a UV detector at 280 nm, which was the best wavelength for the detection of L-Dopa.

### 2.5. In Vivo Nasal Absorption Study

All animal studies performed were approved by the Committee on Animal Research and Ethics guidelines at the Faculty of Pharmacy, Universiti Teknologi MARA. Male Wistar rats, weighing 250–280 g, were fasted for 12 h prior to the experiments, but the rats had free access to water. The rats were anaesthetised by intraperitoneal injection (i.p) of ketamine and xylazine cocktail mixture with a ratio of 60:40 at a dose of 40 mg/kg. A surgical operation for the in vivo nasal absorption study was employed in accordance with the modified method outlined by Hirai et al. (Hirai, Yashiki, and Mima, 1981). Briefly, the rats were placed in the supine position, after which the trachea was exposed via surgical incision to perform tracheal cannulation. The tracheal cannulation was performed in order to maintain respiration. Following that, the trachea leading to the nasal cavity was ligated to prevent the liquid from being exuded from the surgical incision. A polyethene tube was inserted through the oesophagus to the posterior part of the nasal cavity. This step was done to prevent drainage of the applied drug solution into the nasopharynx. After the surgical operation, 20 µL (2.5 mg/kg BW) of sample solution (L-Dopa nanoparticles or L-Dopa drug solution) was administered into the nasal cavity by a micropipette through the nostril. The jugular vein was exposed, and blood samples (0.25 mL) were collected into heparinised syringes at predetermined time intervals from 0 min until 240 min (0, 15 30, 60, 90, 120, 180, 240).

Samples were immediately centrifuged at 12,000 rpm for 5 min to obtain plasma fraction, which was kept in ice until analysis. Concentrations of L-Dopa in rat plasma were determined using the HPLC method detailed in Section 2.4.7. The peak concentration (Cmax) and time to reach peak concentration (Tmax) of these compounds were determined based on the L-dopa plasma profiles over time. The area under the curve (AUC) after nasal administration of these compounds was calculated by the trapezoidal rule from zero to the final sampling time of 240 min.

The absolute bioavailability (BA) of L-dopa in the systemic circulation following the intranasal administration was obtained by comparing the AUC value of intranasal administration with the AUC of intravenous administration. The absolute bioavailability of L-dopa chitosan nanoparticles was calculated as follows:

(2) $$BA\;\left(\%\right)=\frac{AUC_{intranasal}\times Dose_{intravenous}}{AUC_{intravenos}\times Dose_{intranasal}}\times 100\%$$

Based on Equation (2), *BA* refers to absolute bioavailability, *AUC_intranasal_* is the area under the curve following intranasal administration, *Dose_intravenous_*_._ is the dose following intravenous injection, *AUC_intravenous_*_._ is the area under the curve following intravenous administration, and *Dose_intranasal_* is the dose for intranasal administration.

### 2.6. Statistical Analysis

Statistical analysis was performed using GraphPad Prism^®^ version 8.0 (GraphPad Software, San Diego, CA, USA). All experimental results are presented as means ± standard deviation (SD) unless otherwise stated. An unpaired *t*-test was used for the comparison of two cohorts. One-way analysis of variance (ANOVA) was used for the comparison of multiple cohorts. In all cases, *p* < 0.05 is used to denote statistically significance, where *p*-value outputs were 0.033 (*), 0.002 (**), <0.001 (***), and <0.0001 (****).

## 3. Results and Discussion

### 3.1. Characterisation of Nanoparticles

#### 3.1.1. Nanoparticle Size and Dispersity

**Figure 2:**
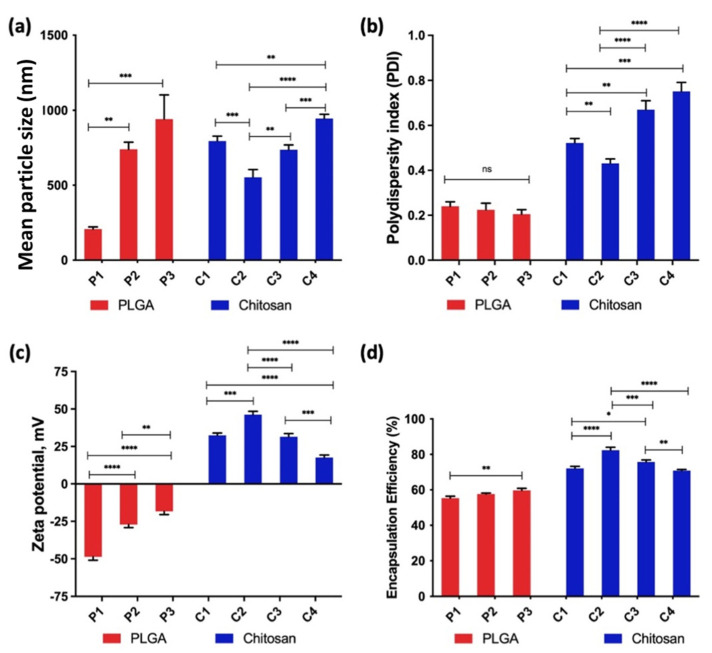
(**a**) Mean particle size (nm), (**b**) uniformity measured as polydispersity index (PDI) for each formulation. (**c**) Effects of different polymer concentrations on the zeta potential and (**d**) encapsulation efficiency of L-Dopa loaded with PLGA and chitosan nanoparticles. Results are expressed as the mean ± SD, *n* = 3. Differences were calculated using one-way ANOVA, followed by Tukey’s post hoc test, and deemed significant at where p-value outputs were 0.033 (*), 0.002 (**), <0.001 (***) and <0.0001 (****).

L-Dopa loaded PLGA nanoparticles were formulated by using a modified W/O/W solvent evaporation technique, while chitosan nanoparticles were manufactured by ionic gelation. Both types of nanoparticle formulations were analysed for particle size and uniformity. Figure 2a shows that increasing PLGA and chitosan concentration had an impact on the size of the nanoparticle produced. With regards to PLGA nanoparticles, P1 (1.25 mg/mL) had the smallest particle size of 207 ± 15 nm, followed by formulation P2 (2.50 mg/mL of PLGA) and P3 (5.00 mg/mL of PLGA) with particle sizes of 739 ± 48 nm and 941 ± 162 nm, respectively. Thus, these results are clearly indicating that there was an increase in nanoparticle size from 200 nm to 940 nm when increasing PLGA concentration. However, all the PLGA nanoparticles formulations showed the same polydispersity index (PDI) (*p* > 0.05). Overall, these findings indicate that PLGA concentration had an effect on the size but not on the PDI during the manufacture of these PLGA nanoparticles. These results are in agreement with those found by Mohan and co-workers, who developed PLGA-polyethylene glycol (PEG) nanoparticles using a solvent dispersion method for enhanced drug targeting to the inflamed intestinal barrier [19]. This observation may be attributed to the propensity of PLGA polymer to clump and coalesce with each other with increasing polymer concentration as the nanoparticles are manufactured via the solvent evaporation technique, thus producing a bigger particle size [20].

On the other hand, it can be seen that polymer concentration also had an impact on the manufacture of L-Dopa loaded chitosan nanoparticles. In contrast to PLGA, chitosan concentration not only had an impact on the particle size of L-Dopa loaded chitosan nanoparticles but also affected the PDI of L-Dopa loaded chitosan nanoparticles. Figure 2a showed that the smallest mean droplet size for chitosan nanoparticles was observed in C2 (553 ± 52 nm), followed by formulation C3, C1, and C4 with the mean droplet sizes of 737 ± 32 nm, 795 ± 33 nm, and 945 ± 29 nm, respectively. Although formulation C2 was prepared using a higher chitosan concentration than C1, it presented a smaller particle size compared to C1. This may be attributed to the low chitosan concentration used in the manufacture of formulation C1 relative to C2. It has been reported that varying chitosan concentration is a convenient way to tune the ionic cross-link density and swelling properties of chitosan/TPP nanoparticles which ultimately affects the overall size of the nanoparticle [21,22,23].

According to these results, it can be concluded that the ratio of sodium TPP and chitosan concentration was optimal for achieving a complete reaction of sodium TPP and chitosan during nanoparticle preparation [24]. However, the low chitosan concentration used in formulation C1 results in excess TPP post-crosslinking which may deposit on the surface of the nanoparticles resulting in an increase in a large nanoparticle relative to C2 [25]. As the chitosan concentration increased to 0.3% *w/v* (C3) and 0.4% *w/v* with fixed TPP concentration, it is hypothesized that there was insufficient TPP to fully cross-link the chitosan polymer per nanoparticle. The presence of uncrosslinked chitosan within these nanoparticles will exhibit a free primary amine group which can form hydrogen bonds with water molecules within the aqueous phase. This hydrogen bond interacting will result in nanoparticle swelling, which culminates in an increase in the hydrodynamic size of the nanoparticle manufactured [26].

The polydispersity of chitosan nanoparticles, as shown in Figure 2b, exhibited a non-linear behaviour with formulation C4 displaying the highest PDI (0.75 ± 0.04) followed by formulation C3 (0.67 ± 0.02), formulation C1 (0.52 ± 0.02), and formulation C2 (0.43 ± 0.04), respectively. It can be seen that chitosan nanoparticles manufactured at higher chitosan concentration showed a higher PDI relative to formulations manufactured at low chitosan concentration. This may be attributed to the interaction between sodium TPP and chitosan. This phenomenon can be explained by the ease of polyanion dispersion within the chitosan network at different polymer concentrations. At low chitosan concentration solution, the polymer exists in an extended conformation due to the repulsion between the positively charged amine groups. This extended conformation confers large spaces within the chitosan network that enabled rapid and uniform dispersion of the polyanion, TPP. The uniformity in TPP dispersion promotes a more homogenous inter- and intra-crosslinking between the protonated amine groups and TPP anions, leading to the production of nanoparticles with a more uniform size distribution. However, as the chitosan concentration increases, the polymer undergoes inter and intramolecular chain entanglement [27]. This entanglement reduces the intermolecular space available for TPP dispersion within the chitosan network. This heterogeneous distribution of TPP polyanions within the chitosan network will lead to an inconsistent degree of cross-linking between nanoparticles, which culminate the production of nanoparticles with a more heterogeneous size distribution as shown by formulation C3 and C4 [28,29,30].

#### 3.1.2. Zeta Potential of Nanoparticles

Zeta potential is a vital parameter that is frequently used to evaluate the stability of the nanoparticles and potential interaction between nanoparticles with mucosal membranes. Nanoparticles with a zeta potential absolute value of 30 mV or above are considered to have good stability with less propensity to agglomerate upon storage [31]. Figure 2c shows the zeta potential values for both PLGA and chitosan nanoparticles. Zeta potential values decreased with increasing PLGA concentrations from P1–P3. The highest zeta potential was observed in formulation P1 (−48.6 ± 2.4 mV), followed by formulation P2 (−27.1 ± 2.1 mV) and formulation P3 (−18.4 ± 2.1 mV). Moreover, all L-dopa loaded PLGA nanoparticles showed a negative zeta potential. These results are in agreement with those previously reported, showing that PLGA nanoparticles tend to exhibit a negative zeta potential when the nanoparticle is reconstituted as a nanosuspension [32,33]. The negative zeta potential is attributed to the ionisation of the terminal carboxyl groups end chain on the PLGA polymer [34]. However, the zeta potential of PLGA nanoparticles is not only affected by the ionisation of the terminal carboxyl group, as the overall zeta potential is also affected by the degree of PLGA coating around the nanoparticle, which in turn is governed by the concentration of PLGA used in the manufacturing of the PLGA nanoparticles [35]. A previous study by [35] showed that high PLGA concentration could increase the coating layers on the polymer surface and shield the charged carboxyl group on the surface of the particles, thus reducing the overall negative zeta potential. Such finding is in agreement with the results observed in the current study and those reported by Huang and Zhang [20], where the zeta potential of PLGA nanoparticles decreased after increasing polymer concentration.

For the L-Dopa loaded chitosan nanoparticles, formulation C2 showed the highest zeta potential (+46.2 mV), followed by formulations C1 (+32.4 mV) and C3 (+31.5 mV), which presented virtually the same values, respectively) (*p* > 0.05) and formulation C4, which displayed the lowest zeta potential (+17.6 ± 1.7 mV). As zeta potential is an indicator of the colloidal stability of nanoparticles, it is frequently suggested that nanoparticles displaying zeta potential values greater than +25 mV or less than −25 mV typically exhibit high degrees of colloidal stability [36]. Accordingly, formulation C2 exhibited the highest colloidal stability. Moreover, the positive zeta potential exhibited by formulations C1–C4 is a cardinal feature of most chitosan-based nanoparticle systems that are fabricated from ionic gelation [37,38]. Chitosan is a semisynthetic material manufactured through the deacetylation of chitin and is comprised of glucosamine (deacetylated monomer) and N-acetyl-glucosamine (acetylated monomer) monomers linked through β-,4 glycosidic bonds. In contrast to PLGA, chitosan contains a lot of free primary amine group, which under colloidal conditions may undergo ionization to form -NH_3_^+^ leading to an overall positive zeta potential.

Nanoparticles exhibiting a positive zeta potential is highly desirable for intranasal mucosal delivery as the positive charge may enhance transport of the protein across the nasal epithelium via increasing the residence time of these nanoparticles in the nasal cavity due to electrostatic interactions with the negatively charged sialic acid residues on mucosal proteins [39]. Such observation contradicts the findings by other researchers, who found that zeta potential was found to be directly proportional to the concentration of chitosan used in the preparation of nanoparticles via ionic gelation [40,41]. In contrast, Zaki et al. found that the chitosan concentration used in the preparation of nanoparticles did not have a significant impact on the overall zeta potential of the nanoparticles for medium and high molecular weight chitosan [42]. However, in this study, we observed that as chitosan concentration increased, the value of zeta potential was decreased. This is attributed to the presence of a higher free amine group on the surface of the chitosan nanoparticle when the formulations were manufactured at higher chitosan concentrations. When the nanoparticles were reconstituted in deionized water post-purification, the presence of the free amine group will cause a rise in the pH of the aqueous media. The rise in pH will cause the zeta potential of the chitosan nanoparticle to decrease, an observation that has been already reported by other researchers [43,44]. As the pH of the reconstituting media rise, less primary amine group will be ionized to form -NH_3_^+^, resulting in lower surface charge on the nanoparticles culminating in an overall lower zeta potential.

#### 3.1.3. Drug Entrapment Efficiency

L-Dopa loaded PLGA nanoparticles were prepared by the W/O/W modified solvent evaporation technique [45]. Figure 2d showed that both formulations P1 (55.28 ± 1.13%) and P2 (57.68 ± 0.53%) showed similar EE. However, formulation P3, which was manufactured with the highest PLGA concentration, showed significantly higher EE (59.65 ± 1.20%) (*p* < 0.05). These results are in agreement with those found in previous studies done by Fu and researchers [46,47]. Drug loading into nanoparticles via W/O/W modified solvent evaporation technique is considered one of the most challenging methods in achieving high drug EE [47,48]. Using this method, EE of nanoparticles and microparticles would be dependent on the drug partition coefficient between the internal and external phases of the emulsion [49]. Therefore, the extent of drug loading is dependent on the rate of nanoparticle solidification. Zhu et al. have shown that a faster solidification rate may to some degree enhance the EE of the nanoparticle system [50]. In this study, it can be seen that increasing PLGA concentration did, to some degree, improve the encapsulation efficiency of the nanoparticulate system. This is because with increasing polymer concentration, the rate of polymer precipitation and solidification from the oil phase was enhanced under the same temperature and shear strength [46].

L-Dopa loaded chitosan nanoparticles displayed higher EE in comparison with PLGA nanoparticles. This could be explained by the quick cross-linking between sodium TPP and chitosan, leading to a quicker nanoparticle formation and solidification, resulting in a higher EE [51]. For L-Dopa loaded chitosan nanoparticles, formulation C2, which showed the smallest mean droplet size (553 ± 52 nm), exhibited the highest drug content (82.38 ± 1.63%), followed by formulation C3 (75.73 ± 1.14%), formulation C1 (72.07% ± 1.21) and formulation C4 (70.84% ± 0.62%), respectively. This higher EE exhibited by formulation C2 may be attributed to the quicker and higher degree of cross-linking between chitosan and sodium TPP at this chitosan/sodium TPP ratio in comparison with the rest of the formulations [52]. The high degree of cross-linking at this ratio of chitosan/sodium TPP not only promoted smaller nanoparticle formation but also contributed to faster nanoparticle solidification, which is pertinent in mitigating the drug from partitioning out of the nanoparticles and into the aqueous phase [24].

A further increase in the chitosan concentration (C2-C4) with the same sodium TPP concentration resulted in a significant decrease in the drug EE (*p* < 0.05), as shown in Figure 2d. This may be attributed to a lower degree of chitosan cross-linking when increasing polymer concentration under the same sodium TPP concentration. The lower degree of cross-linking led to a slower rate of nanoparticle formation, which in turn resulted in some L-Dopa to partition out in the aqueous phase leading to a decrease in EE. L-Dopa loaded chitosan nanoparticles displayed better EE than the ones made from PLGA. Following the nanoparticle characterisation study, L-Dopa loaded chitosan nanoparticles were selected over PLGA nanoparticles for further characterisation along with in vivo evaluation. This is because the chitosan nanoparticles formulation displayed higher drug loading and entrapment efficiency relative to L-Dopa loaded PLGA nanoparticles. In order to further elucidate the properties of L-Dopa loaded chitosan nanoparticles, morphology studies were conducted using TEM and SEM.

#### 3.1.4. Morphology of L-Dopa Loaded Chitosan Nanoparticle Characterisation

**Figure 3:**
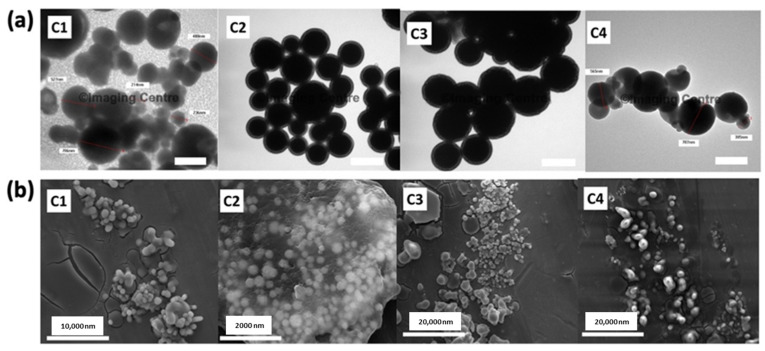
(**a**) TEM image of L-Dopa loaded chitosan nanoparticles for formulation C1–C4. Scale bar: 500,000 nm (**b**) SEM image of L-Dopa loaded chitosan nanoparticles for formulation C1–C4. Scale bar for respective image is embedded in the image.

TEM in tandem with SEM was used to study the shape, surface appearance, and structural morphology of chitosan polymeric nanoparticles. TEM and SEM oictures of L-Dopa loaded chitosan nanoparticles (C1–C4) are shown in Figure 3. All the performed L-Dopa loaded chitosan nanoparticles presented a spherical shape with a smooth surface. Nanoparticles in formulations C1 and C3 were spherical but displayed low size uniformity. There was some degree of aggregations between the nanoparticles within the formulations C1 and C3, resulting in the formation of different groups of clumped particulates, and in some instances, these nanoparticles have coalesced into nanoparticles with larger diameters. Moreover, both formulations, C1 and C3, presented almost the same particle size with an average diameter of ≈750 nm. The aggregation of chitosan nanoparticles in formulation C1 may be attributed to the presence of free sodium TPP, which is in excess of the reaction between sodium TPP and chitosan. Under this condition, the presence of free sodium TPP would bind to the surface of the nanoparticle and behave as a bridging ion, thus promoting nanoparticle aggregation, as suggested by Huang and Lapitsky [53].

In contrast, formulation C2 showed a smaller particle size and more uniform size distribution. These results are in agreement with those presented in Figure 2, indicating that formulation C2 displayed the lowest PDI and the smallest average particle size out of all four evaluated formulations. Additionally, nanoparticles from formulation C2 were enclosed in a chitosan gel, unlike the rest of the chitosan formulations. It is suggested that this gel structure confers mucoadhesive properties that enhance the residence time of the formulation on the mucosal lining. Such properties are pertinent in enhancing the absorption of the nanoparticles across the nasal mucosa [53].

Furthermore, it can also be seen from Figure 3 that formulation C4 displayed the largest particle size, evidenced by the presence of large particles. Such poor uniformity in particle size shown by formulation C4 is corroborated by the result displayed in Figure 2 that showed that this formulation displayed the highest PDI out of the four formulations developed. The higher particle aggregation in the formulation C4 may be attributed to the presence of additional chitosan layers on the nanoparticles when increasing polymer concentration. The existence of these additional chitosan layers on the nanoparticles also results in the presence of free -NH_2_ which have not been cross-linked with sodium TPP during nanoparticle preparation. When the nanoparticles for these formulations are reconstituted in deionised water post-purification, the presence of these free -NH_2_ raises the pH of the reconstitution media. The rise in pH has been shown by Huang and Lapitsky to induce the aggregation of chitosan nanoparticles [53]. This was further confirmed by the work of Saini et al., who showed that with an increase in the pH, the charge on the chitosan nanoparticle is reduced, resulting in poor colloidal stabilisation via electrostatic repulsion, which induces nanoparticle aggregation [54].

Overall, the TEM and SEM images showed minimal nanoparticle aggregation in formulation C2. This is because the ratio of ionic cross-linking agents, sodium TPP and chitosan were at an optimal ratio to promote sufficient polymer cross-linking with minimal presence of free TPP and -NH_2_ on chitosan post-manufacture. The presence of a minimal concentration of free TPP and unreacted -NH_2_ on chitosan helped mitigate nanoparticle aggregation [53]. However, when the concentration of chitosan used was increased any further, we observed large aggregations within the nanoparticle systems, as shown in formulation C3 and C4, which could not be mitigated even though Tween^®^ 80 was added as a suspending medium to promote nanoparticle stabilisation [55]. This indicated that the ratio of chitosan:sodium TPP played a pivotal role not only on the overall size and EE of the system but ultimately on the stability of the nanoparticles. From the results above, formulation C2 showed the smallest size and the best particle size distribution for L-Dopa loaded chitosan nanoparticles. Therefore, formulation C2 was selected for further characterisation using XRD and FTIR.

#### 3.1.5. Powder X-ray Diffraction Analysis

**Figure 4:**
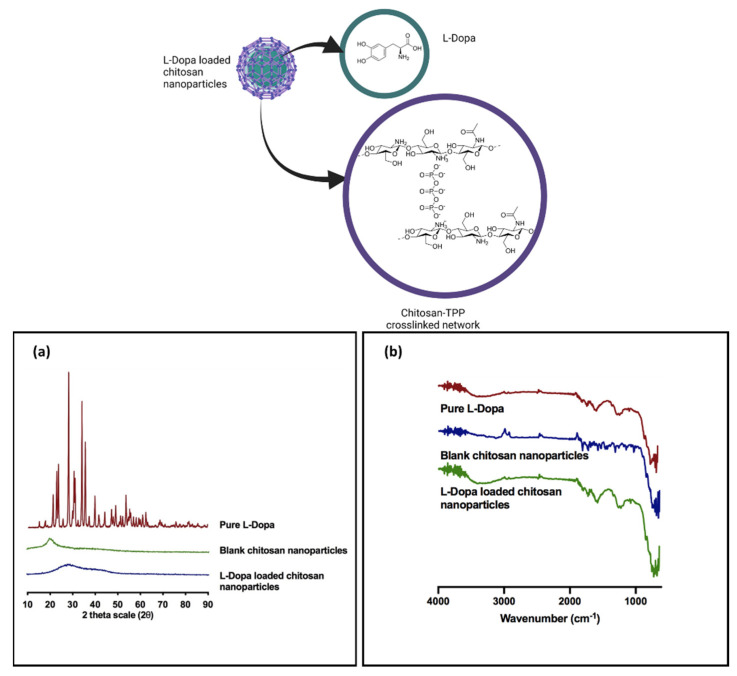
Physical characterisation of L-Dopa loaded chitosan nanoparticles. (**a**) Diffractogram of pure L-Dopa, blank chitosan nanoparticles and L-Dopa loaded chitosan nanoparticles. (**b**) FTIR spectrum of pure L-Dopa, blank chitosan nanoparticles and L-Dopa loaded chitosan nanoparticles.

XRD analysis was conducted on chitosan nanoparticle formulation C2 in order to elucidate the solid state of the drug that is present within the nanoparticles. The X-ray diffraction patterns for L-Dopa are presented in Figure 4a. The diffractogram showed noticeable crystalline peaks at 2θ of 17.95°, 18.40°, 21.25°, 22.75°, 24.95° and 25.90° accordingly with intensity ranging from 2000 cps to 4500 cps. The presence of these peaks at such intensity are cardinal markers that confirm the highly crystalline state of L-Dopa [56,57].

The XRD diffractogram of blank chitosan showed a typical amorphous diffractogram without any apparent crystalline peaks. When the chitosan nanoparticles were loaded, L-Dopa was present in an amorphous state, as evidenced by the absence of any crystalline peak in Figure 4a. This suggests that the method of preparing the chitosan nanoparticle via ionic gelation caused L-Dopa to change its state from crystalline to an amorphous state. The amorphous state of the L-Dopa in chitosan nanoparticles may be attributed to the bulky yet dense network structure of penetrating polymer chains cross-linked with sodium TPP counterions that prevent the L-Dopa from recrystallisation [58]. Alternatively, the presence of amine and hydroxyl groups on the chitosan polymer may form interaction via hydrogen bonding and ion-dipole between the L-Dopa and the polymer that prevent drug molecules from aggregating and recrystallising [59]. The presence of L-Dopa in an amorphous state is highly desirable as the drug would have improved solubility that would promote faster drug release from the nanoparticle upon administration.

#### 3.1.6. Fourier Transform Infrared Analysis

FTIR analysis was performed to evaluate any potential interactions between L-Dopa and blank chitosan nanoparticles within the samples (Figure 4b). The spectra for L-Dopa exhibit peaks at 3183.59 cm^−1^, 1651.89 cm^−1^ and 1451.22 cm^−1^ which are characteristics of the pure drug. The peak at 1651.89 cm^−1^ represents the di-substituted aromatic ring, while the peak at 1451.22 cm^−1^ represents the O-H stretch from the carboxylic acid group on the drug. Moreover, the peak at 3183.59 cm^−1^ indicates the amine group on the drug molecule [57].

Blank chitosan nanoparticles showed characteristic peaks at 1375.44 cm^−1^, 1584.46 cm^−1^ and 3286.56 cm^−1^. The peak at 3286.56 cm^−1^ represents the amine group and O-H stretching on the biopolymer, as well as the intramolecular hydrogen bonds, while the peak at 1584.46 cm-1 corresponds to the N-H bending of the primary amine [60]. The peak at 1150.07 cm^−1^ can be attributed to the asymmetric stretching of the C-O-C bridge between the repeating units within the chitosan biopolymer [61]. Moreover, the CH3 symmetrical deformations were confirmed by the presence of a band at 1375.44 cm−1 [61]. The FTIR analysis revealed that L-Dopa peaks could be found in the IR spectrum of the L-Dopa loaded chitosan nanoparticle samples. Additionally, no new peaks or peak shifts were obtained, suggesting that no chemical reaction took place during the ionic gelation method used to prepare the drug-loaded chitosan nanoparticles.

### 3.2. In Vivo Intranasal Delivery Study

Intranasal administration has garnered considerable attention among pharmaceutical scientists as a potential route for achieving faster absorption with optimal bioavailability. Indeed, the delivery of L-Dopa via the intranasal route would be of great advantage relative to oral delivery as more than 80% of patients with Parkinson’s disease suffers dysphagia at some point during the course of the disease [5]. In the current work, L-Dopa loaded chitosan nanoparticles were prepared and characterised, followed by in vivo evaluation in rats. Based on the characterisation study, formulation C2 was selected for in vivo intranasal delivery as this formulation displayed the highest drug EE and zeta potential. The positive charge on the nanoparticle may enhance the transport of the system across the nasal epithelium via increasing the residence time of the nanoparticle in the nasal cavity. Such extension in residence time may be due to the electrostatic interactions between the nanoparticles with the negatively charged sialic acid residues present on the mucous along the nasal linings [39].

Upon intranasal administration into male Wistar rats, plasma samples were collected at defined time points in order to evaluate the absorption and bioavailability of L-Dopa loaded chitosan nanoparticles following intranasal administration relative to the control group. The control group in this study received intranasal administration of unmodified L-Dopa as a drug solution. In addition, IV injections were administered to a separate group of rats to allow us to evaluate the absolute bioavailability for respective treatment groups. The pharmacokinetic parameters evaluated were the area under the curve (AUC) from time 0 to 240 min, maximum drug concentration in plasma (C_max_) and maximum time to reach maximum drug concentration in plasma (T_max_). Table 3 shows AUC, C_max_ and T_max_ values following intranasal administration of either 2.5 mg/kg of L-Dopa loaded chitosan nanoparticles or 2.5 mg/kg of drug solution. From the table, AUC_0–240_ was determined using the linear trapezoidal rule. The mean AUC value for L-Dopa drug solution was 6494.582 ± 688.38, while L-Dopa loaded chitosan nanoparticle was 11,054.16 ± 1381.5. The AUC value of L-Dopa loaded chitosan nanoparticles was significantly (*p* < 0.05) higher (by two-fold) than that of intranasal administration of L-Dopa drug solution alone. The data showed that chitosan nanoparticles were capable of enhancing the absorption of L-Dopa across the nasal epithelium enabling the drug to bypass first-pass metabolism leading to enhanced bioavailability.

**Figure 5:**
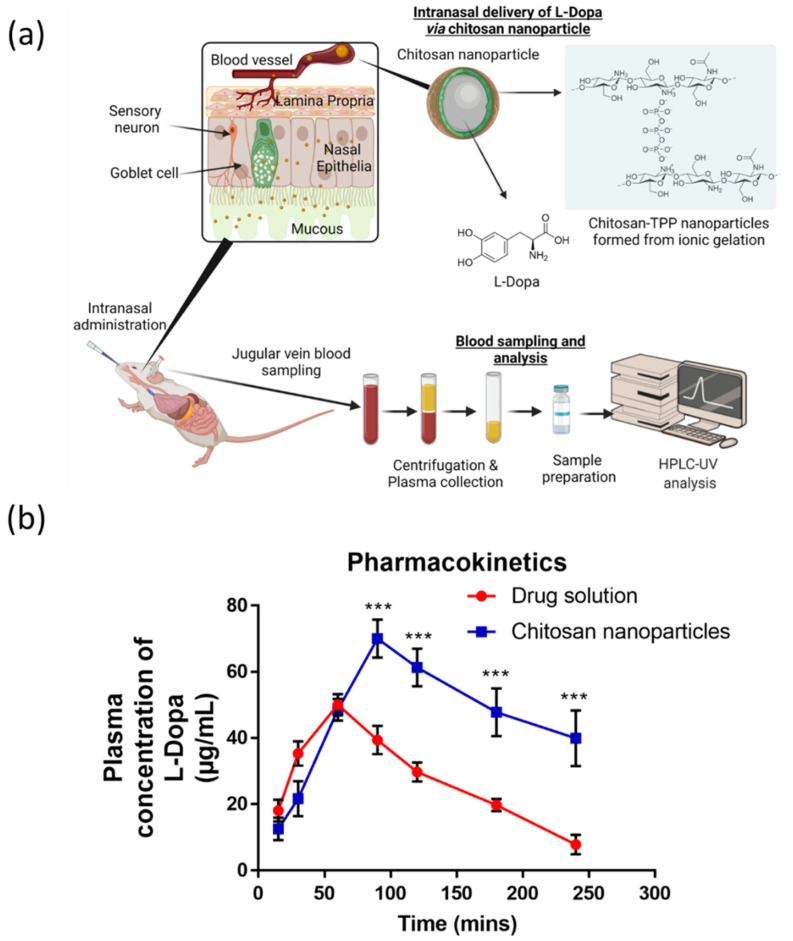
(**a**) Schematic illustrating intranasal administration of L-Dopa loaded chitosan nanoparticles into male Wistar rat. (**b**) Pharmacokinetic profile of 2.5 mg/kg L-Dopa drug solution and 2.5 mg/kg L-Dopa loaded chitosan nanoparticles (Formulation C2) after intranasal administration. Results are expressed as the mean ± SD, n = 4. Differences were calculated using one-way ANOVA, followed by Tukey’s post hoc test, and deemed significant at where p-value outputs were <0.001 (***).

The T_max_ for L-Dopa administered as a drug solution via the intranasal route was 60 min, indicating rapid absorption of the drug into the systemic circulation. This T_max_ was much faster than the reported T_max_ for L-Dopa that was given via oral administration, which is 90 min (Luinstra et al. (2019). After reaching the peak plasma concentration, L-Dopa concentration declined drastically due to the half-life of the drug, which is between 45–90 min. In addition, any drug solution that is present along nasal mucosal after intranasal administration will be eliminated rapidly eliminated due to mucociliary clearance along the nasal cavity [62]. In contrast, there is a delay in time to reach the peak concentration, T_max_ by 30 min for L-Dopa loaded chitosan nanoparticles, as shown in Table 3 and Figure 5b. This may be attributed to the size of the nanoparticle, which is far larger than the free drug molecule, thus necessitating a longer time for the nanoparticles to diffuse across the nasal mucosa before being absorbed into systemic circulation, thus delaying the time needed to reach T_max_.

Based on Figure 4b, the plasma concentration of L-Dopa loaded chitosan nanoparticles was significantly higher (*p* < 0.05) compared to intranasal administration of L-Dopa drug solution at time points 90, 120, 180 and 240 min. As shown in Table 3, the mean value of absolute bioavailability (F%) for L-Dopa drug solution and L-Dopa loaded chitosan nanoparticles was 26.56% and 45.20%, respectively. From the results, we can conclude that L-Dopa loaded chitosan nanoparticles formulation has a higher bioavailability compared to L-Dopa drug solution by two-fold. The mucoadhesive properties of chitosan also endow the nanoparticles with a longer residence time along the nasal mucosa [63]. This innate property of chitosan prolonged the period that L-Dopa can be absorbed along the nasal mucosa leading to improved drug exposure as evidenced from the higher AUC, as shown in Figure 4b. The mucoadhesive properties of the nanoparticles also enable sustained-release of the drug, thus mitigating plasma L-Dopa level from dropping drastically, as evident in Figure 4b and Figure S1 relative to intranasal administration of drug solution.

## 4. Conclusions

In conclusion, the current work highlights the fabrication, characterisation and evaluation of L-Dopa loaded polymeric nanoparticles for the treatment of Parkinson’s disease. PLGA and chitosan were used as a polymer matrix for the development of these nanoparticles. PLGA nanoparticles (formulations P1, P2 and P3) were prepared using solvent evaporation technique, while chitosan nanoparticles (formulation C1, C2, C3 and C4) were prepared by ionic gelation method with sodium TPP as a cross-linking agent to entrap the payload. Characterisation studies were carried out to determine the particle size distribution, zeta potential, morphology and drug content of the polymeric nanoparticles. Based on the findings, L-Dopa loaded chitosan nanoparticles (formulation C2) with a particle size of 553 ± 52 nm and zeta potential of 46.2 ± 2.3 has the highest EE (82.38%). Additionally, XRD analysis showed that L-Dopa was present in an amorphous state within the chitosan nanoparticles. FTIR analysis demonstrated that there was no chemical alteration to the drug upon loading into chitosan nanoparticle. Finally, an in vivo study was conducted to evaluate the pharmacokinetic profile of L-Dopa nanoparticles relative to drug solution following intranasal administration. The pharmacokinetic profile showed that the AUC value for L-Dopa loaded nanoparticles (Formulation C2) was significantly higher *(p <* 0.05) by almost two-fold relative to the L-Dopa drug solution. However, L-Dopa loaded chitosan nanoparticles showed a delayed T_max_ of 90 min relative to the L-Dopa drug solution that has a T_max_ of 60 min. Nevertheless, the L-Dopa loaded chitosan exhibited a higher drug release profile due to the mucoadhesive properties of chitosan, thus mitigating the L-Dopa plasma level from dropping drastically. Should this system be translated into clinical practice, intranasal delivery of L-Dopa loaded chitosan nanoparticles may be a suitable treatment alternative to oral administration for patients with Parkinson’s disease who frequently suffer dysphagia.

## Figures and Tables

**Table 1:** Formulation composition of L-Dopa loaded PLGA nanoparticles.

Formulation	L-Dopa (mg/mL)	PLGA (mg/mL)	PVA (% *w/v*)
P1	1	1.25	0.2
P2	1	2.50	0.2
P3	1	5.00	0.2

**Table 2:** Formulation composition of L-Dopa loaded chitosan nanoparticles.

Formulation	L-Dopa (% *w/v*)	Chitosan (% *w/v*)	Tween^®^ 80 (% *w/v*)	Sodium TPP (% *w/v*)
C1	0.1	0.1	0.5	0.1
C2	0.1	0.2	0.5	0.1
C3	0.1	0.3	0.5	0.1
C4	0.1	0.4	0.5	0.1

**Table 3:** Pharmacokinetic profiles of 2.5 mg/kg L-dopa and 2.5 mg/kg L-dopa loaded chitosan nanoparticles (formulation C2) after intranasal administration. Results are expressed as the mean ± S.E. of 3 replicates; * *p* < 0.05.

Sample	C_max_ (µg/mL)	T_max_ (min)	Area under the Curve (AUC)	Absolute Bioavailability, F (%)
L-dopa	50.018 ± 3.25	60	6494.582 ± 272.04	26.56 ± 1.11
L-dopa loaded chitosan NP	70.008 ± 5.77 *	90	11,054.160 ± 1153.50 *	45.20 ± 4.72 *

## Data Availability

Data is contained within the article and supplementary material.

## Supplementary Materials

The following supporting information can be downloaded at: https://www.mdpi.com/article/10.3390/ph15030370/s1, Figure S1: release profile of L-Dopa from chitosan nanoparticles (C2) when under physiological pH of nasal lining (pH 6.0) at 37 °C.

## References

[1] Pagliaro L.A., Pagliaro A.M., Pagliaro L.A., Pagliaro A.M. (2020). Levodopa⋆ [L-Dopa]. PNDR: Psychologists’ Neuropsychotropic Drug Reference.

[2] Lerner R.P., Francardo V., Fujita K., Bimpisidis Z., Jourdain V.A., Tang C.C., Dewey S.L., Chaly T., Cenci M.A., Eidelberg D. (2017). Levodopa-induced abnormal involuntary movements correlate with altered permeability of the blood-brain-barrier in the basal ganglia. Sci. Rep..

[3] Tambasco N., Romoli M., Calabresi P. (2017). Levodopa in Parkinson’s Disease: Current Status and Future Developments. Curr. Neuropharmacol..

[4] Homayun B., Lin X., Choi H.-J. (2019). Challenges and Recent Progress in Oral Drug Delivery Systems for Biopharmaceuticals. Pharmaceutics.

[5] Suttrup I., Warnecke T. (2016). Dysphagia in Parkinson’s Disease. Dysphagia.

[6] Erdő F., Bors L.A., Farkas D., Bajza Á., Gizurarson S. (2018). Evaluation of Intranasal Delivery Route of Drug Administration for Brain Targeting. Brain Res. Bull..

[7] Davis S.S., Illum L. (2003). Absorption Enhancers for Nasal Drug Delivery. Clin. Pharmacokinet..

[8] Lee Y.H., Kim K.H., Yoon I.K., Lee K.E., Chun I.K., Rhie J.Y., Gwak H.S. (2014). Pharmacokinetic evaluation of formulated levodopa methyl ester nasal delivery systems. Eur. J. Drug Metab. Pharmacokinet..

[9] Tang S., Wang A., Yan X., Chu L., Yang X., Song Y., Sun K., Yu X., Liu R., Wu Z. (2019). Brain-targeted intranasal delivery of dopamine with borneol and lactoferrin co-modified nanoparticles for treating Parkinson’s disease. Drug Deliv..

[10] Bustamante-Marin X.M., Ostrowski L.E. (2017). Cilia and Mucociliary Clearance. Cold Spring Harb. Perspect. Biol..

[11] Zainuddin S.Z., Hamid K.A. (2021). Chitosan-Based Oral Drug Delivery System for Peptide, Protein and Vaccine Delivery. Chitin and Chitosan—Physicochemical Properties and Industrial Applications [Working Title].

[12] Yu S., Xu X., Feng J., Liu M., Hu K. (2019). Chitosan and chitosan coating nanoparticles for the treatment of brain disease. Int. J. Pharm..

[13] Comoglu T., Arisoy S., Atalay O., Onal D., Pehlivanoglu B. (2020). Development and in vivo application of levodopa loaded polylactic co- gylicolic acid (PLGA) nanoparticles for nazal brain drug delivery. Park. Relat. Disord..

[14] Arisoy S., Sayiner O., Comoglu T., Önal D., Atalay O., Pehlivanoglu B. (2020). In vitro and in vivo evaluation of levodopa-loaded nanoparticles for nose to brain delivery. Pharm. Dev. Technol..

[15] Sharma S., Lohan S., Murthy R.S.R. (2013). Formulation and characterization of intranasal mucoadhesive nanoparticulates and thermo-reversible gel of levodopa for brain delivery. Drug Dev. Ind. Pharm..

[16] Budhian A., Siegel S.J., Winey K.I. (2007). Haloperidol-loaded PLGA nanoparticles: Systematic study of particle size and drug content. Int. J. Pharm..

[17] Rathi Mahesh C., Girish Sailor A.K., Seth S.P.C. (2013). Development and characterization of quetiapine loaded chitosan nanoparticle. Pharma Sci. Monit..

[18] Mohd Z.A., Khuriah A.H. A Validated High-Performance Liquid Chromatographic Method for The Determination of Levodopa in Rat Plasma & Its Application in Pharmacokinetic Studies. Proceedings of the 2012 IEEE Symposium on Business, Engineering and Industrial Applications.

[19] Mohan L.J., McDonald L., Daly J.S., Ramtoola Z. (2020). Optimising PLGA-PEG Nanoparticle Size and Distribution for Enhanced Drug Targeting to the Inflamed Intestinal Barrier. Pharmaceutics.

[20] Huang W., Zhang C. (2018). Tuning the Size of Poly(lactic-co-glycolic Acid) (PLGA) Nanoparticles Fabricated by Nanoprecipitation. Biotechnol. J..

[21] Sawtarie N., Cai Y., Lapitsky Y. (2017). Preparation of chitosan/tripolyphosphate nanoparticles with highly tunable size and low polydispersity. Colloids Surfaces B Biointerfaces.

[22] Rampino A., Borgogna M., Blasi P., Bellich B., Cesàro A. (2013). Chitosan nanoparticles: Preparation, size evolution and stability. Int. J. Pharm..

[23] Cai Y., Lapitsky Y. (2017). Analysis of chitosan/tripolyphosphate micro- and nanogel yields is key to understanding their protein uptake performance. J. Colloid Interface Sci..

[24] Lazaridou M., Christodoulou E., Nerantzaki M., Kostoglou M., Lambropoulou D.A., Katsarou A., Pantopoulos K., Bikiaris D.N. (2020). Formulation and In-Vitro Characterization of Chitosan-Nanoparticles Loaded with the Iron Chelator Deferoxamine Mesylate (DFO). Pharmaceutics.

[25] Piani L., Papo A. (2013). Sodium Tripolyphosphate and Polyphosphate as Dispersing Agents for Alumina Suspensions: Rheological Characterization. J. Eng..

[26] Al-Nemrawi N.K., Alsharif S.S.M., Dave R.H. (2018). Preparation of chitosan-tpp nanoparticles: The influence of chitosan polymeric properties and formulation variables. Int. J. Appl. Pharm..

[27] Dong Y., Ng W.K., Shen S., Kim S., Tan R.B. (2013). Scalable ionic gelation synthesis of chitosan nanoparticles for drug delivery in static mixers. Carbohydr. Polym..

[28] Qun G., Ajun W. (2006). Effects of molecular weight, degree of acetylation and ionic strength on surface tension of chitosan in dilute solution. Carbohydr. Polym..

[29] Berger J., Reist M., Mayer J., Felt O., Peppas N., Gurny R. (2004). Structure and interactions in covalently and ionically crosslinked chitosan hydrogels for biomedical applications. Eur. J. Pharm. Biopharm..

[30] Fan W., Yan W., Xu Z., Ni H. (2012). Formation mechanism of monodisperse, low molecular weight chitosan nanoparticles by ionic gelation technique. Colloids Surfaces B Biointerfaces.

[31] Lowry G.V., Hill R.J., Harper S., Rawle A.F., Hendren C.O., Klaessig F., Nobbmann U., Sayre P., Rumble J. (2016). Guidance to improve the scientific value of zeta-potential measurements in nanoEHS. Environ. Sci. Nano.

[32] Al Meslmani B.M., Mahmoud G.F., Bakowsky U. (2017). Development of expanded polytetrafluoroethylene cardiovascular graft platform based on immobilization of poly lactic- co -glycolic acid nanoparticles using a wet chemical modification technique. Int. J. Pharm..

[33] Nicolete R., dos Santos D.F., Faccioli L.H. (2011). The uptake of PLGA micro or nanoparticles by macrophages provokes distinct in vitro inflammatory response. Int. Immunopharmacol..

[34] Chiu H.I., Samad N.A., Fang L., Lim V. (2021). Cytotoxicity of targeted PLGA nanoparticles: A systematic review. RSC Adv..

[35] Parveen S., Sahoo S.K. (2011). Long circulating chitosan/PEG blended PLGA nanoparticle for tumor drug delivery. Eur. J. Pharmacol..

[36] Joseph E., Singhvi G. (2019). Multifunctional Nanocrystals for Cancer Therapy: A Potential Nanocarrier.

[37] Patel N., Zariwala M.G., Al-Obaidi H. (2020). Fabrication of Biopolymer Based Nanoparticles for the Entrapment of Chromium and Iron Supplements. Processes.

[38] Karimi M., Avci P., Ahi M., Gazori T., Hamblin M.R., Naderi-Manesh H. (2014). Evaluation of Chitosan-Tripolyphosphate Nanoparticles as a p-ShRNA Delivery Vector: Formulation, Optimization and Cellular Uptake Study. J. Nanopharm. Drug Deliv..

[39] Migliore M.M., Vyas T.K., Campbell R.B., Amiji M.M., Waszczak B.L. (2010). Brain delivery of proteins by the intranasal route of administration: A comparison of cationic liposomes versus aqueous solution formulations. J. Pharm. Sci..

[40] Ing L.Y., Zin N.M., Sarwar A., Katas H. (2012). Antifungal Activity of Chitosan Nanoparticles and Correlation with Their Physical Properties. Int. J. Biomater..

[41] Pawar V.K., Asthana S., Mishra N., Chaurasia M., Chourasia M.K. (2013). Chitosan coated hydroxypropyl methylcellulose-ethylcellulose shell based gastroretentive dual working system to improve the bioavailability of norfloxacin. RSC Adv..

[42] Zaki S.S.O., Ibrahim M.N., Katas H. (2015). Particle Size Affects Concentration-Dependent Cytotoxicity of Chitosan Nanoparticles towards Mouse Hematopoietic Stem Cells. J. Nanotechnol..

[43] Huang G.-Q., Xiao J.-X., Jia L., Yang J. (2014). Complex Coacervation of O-Carboxymethylated Chitosan and Gum Arabic. Int. J. Polym. Mater. Polym. Biomater..

[44] Swain S.K., Dey R.K., Islam M., Patel R., Jha U., Patnaik T., Airoldi C. (2009). Removal of Fluoride from Aqueous Solution Using Aluminum-Impregnated Chitosan Biopolymer. Sep. Sci. Technol..

[45] Iqbal M., Zafar N., Fessi H., Elaissari A. (2015). Double emulsion solvent evaporation techniques used for drug encapsulation. Int. J. Pharm..

[46] Fu X., Ping Q., Gao Y. (2005). Effects of formulation factors on encapsulation efficiency and release behaviourin vitroof huperzine A-PLGA microspheres. J. Microencapsul..

[47] Ray S., Mishra A., Mandal T.K., Sa B., Chakraborty J. (2015). Optimization of the process parameters for the fabrication of a polymer coated layered double hydroxide-methotrexate nanohybrid for the possible treatment of osteosarcoma. RSC Adv..

[48] Alshamsan A. (2014). Nanoprecipitation Is More Efficient than Emulsion Solvent Evaporation Method to Encapsulate Cucurbitacin I in PLGA Nanoparticles, Elsevier Enhanced Reader. Saudi Pharm. J..

[49] Al-Maaieh A., Flanagan D.R. (2001). Salt and cosolvent effects on ionic drug loading into microspheres using an O/W method. J. Control. Release.

[50] Zhu H.L.J.K.J. (2001). Preparation and characterization of hCG-loaded polylactide or poly(lactide-co-glycolide) microspheres using a modified water-in-oil-in-water (w/o/w) emulsion solvent evaporation technique. J. Microencapsul..

[51] Pedroso-Santana S., Fleitas-Salazar N. (2020). Ionotropic gelation method in the synthesis of nanoparticles/microparticles for biomedical purposes. Polym. Int..

[52] Xu Y., Du Y. (2003). Effect of Molecular Structure of Chitosan on Protein Deli v Ery Properties of Chitosan Nanoparticles. Int. J. Pharm..

[53] Huang Y., Lapitsky Y. (2017). On the kinetics of chitosan/tripolyphosphate micro- and nanogel aggregation and their effects on particle polydispersity. J. Colloid Interface Sci..

[54] Saini R., Srivastava A., Gupta P., Das K. (2011). pH dependent reversible aggregation of Chitosan and glycol-Chitosan stabilized silver nanoparticles. Chem. Phys. Lett..

[55] Biró E., Németh Á.S., Sisak C., Feczkó T., Gyenis J. (2008). Preparation of chitosan particles suitable for enzyme immobilization. J. Biochem. Biophys. Methods.

[56] Luinstra M., Grasmeijer F., Hagedoorn P., Moes J.R., Frijlink H.W., de Boer A.H. (2015). A levodopa dry powder inhaler for the treatment of Parkinson’s disease patients in off periods. Eur. J. Pharm. Biopharm..

[57] Ledeti I., Bolintineanu S., Vlase G., Circioban D., Ledeti A., Vlase T., Suta L.-M., Caunii A., Murariu M. (2017). Compatibility study between antiparkinsonian drug Levodopa and excipients by FTIR spectroscopy, X-ray diffraction and thermal analysis. J. Therm. Anal..

[58] Qi L., Xu Z., Jiang X., Hu C., Zou X. (2004). Preparation and antibacterial activity of chitosan nanoparticles. Carbohydr. Res..

[59] Kestur U.S., Taylor L.S. (2010). Role of polymer chemistry in influencing crystal growth rates from amorphous felodipine. CrystEngComm.

[60] Lim S.-H., Hudson S.M. (2004). Synthesis and antimicrobial activity of a water-soluble chitosan derivative with a fiber-reactive group. Carbohydr. Res..

[61] Fernandes Queiroz M., Melo K.R.T., Sabry D.A., Sassaki G.L., Rocha H.A.O. (2015). Does the Use of Chitosan Contribute to Oxalate Kidney Stone Formation?. Mar. Drugs.

[62] Agu R.U. (2016). Challenges in nasal drug absorption: How far have we come?. Ther. Deliv..

[63] Cortés H., Alcalá-Alcalá S., Caballero-Florán I.H., Bernal-Chávez S.A., Ávalos-Fuentes A., González-Torres M., Carmen M.G.-D., Figueroa-González G., Reyes-Hernández O.D., Floran B. (2020). A Reevaluation of Chitosan-Decorated Nanoparticles to Cross the Blood-Brain Barrier. Membranes.

