# Supplementary material for: Design and Development of Levodopa Loaded Polymeric Nanoparticles for Intranasal Delivery
Source: Pharmaceuticals (Basel). 2022 Mar 18;15(3):370. doi: 10.3390/ph15030370 (PMC8951268; doi:10.3390/ph15030370)
Supplement: Supplementary file 1 [file pharmaceuticals-15-00370-s001.zip › pharmaceuticals-1597757-supplementary.pdf]

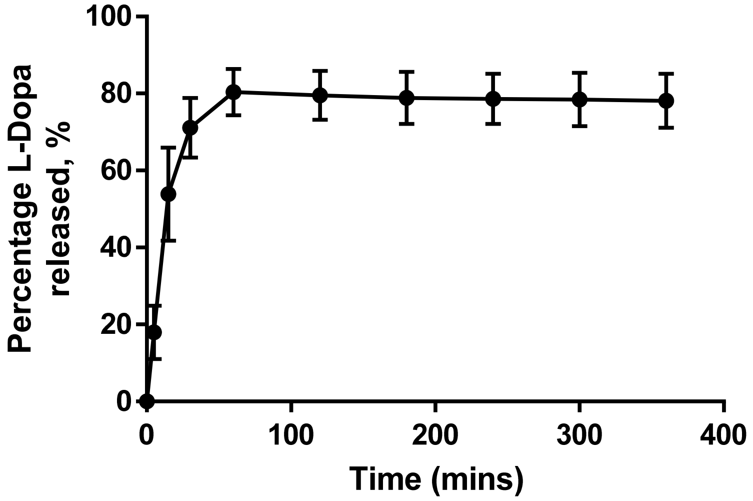

Figure S1 release profile of L-Dopa from chitosan nanoparticles (C2) when under physiological pH of nasal lining (pH 6.0) at 37°C. Data expressed as mean ± SEM (n=3)
